# A population-based study of anxiety as a precursor for depression in childhood and adolescence

**DOI:** 10.1186/1471-244X-4-43

**Published:** 2004-12-13

**Authors:** Frances Rice, Marianne BM van den Bree, Anita Thapar

**Affiliations:** 1 Department of Psychological Medicine, School of Medicine, Cardiff University, Cardiff, UK; 2 School of Psychology, Cardiff University, Cardiff, UK

**Keywords:** Anxiety, depression, child, twin, longitudinal, genetic

## Abstract

**Background:**

Anxiety and depression co-occur in children and adolescents with anxiety commonly preceding depression. Although there is some evidence to suggest that the association between early anxiety and later depression is explained by a shared genetic aetiology, the contribution of environmental factors is less well examined and it is unknown whether anxiety itself is a phenotypic risk factor for later depression. These explanations of the association between early anxiety and later depression were evaluated.

**Methods:**

Anxiety and depressive symptoms were assessed longitudinally in a U.K. population-based sample of 676 twins aged 5–17 at baseline. At baseline, anxiety and depression were assessed by parental questionnaire. Depression was assessed three years later by parental and adolescent questionnaire.

**Results:**

Shared genetic effects between early anxiety and later depression were found. A model of a phenotypic risk effect from early anxiety on later depression provided a poor fit to the data. However, there were significant genetic effects specific to later depression, showing that early anxiety and later depression do not index entirely the same genetic risk.

**Conclusions:**

Anxiety and depression are associated over time because they share a partly common genetic aetiology rather than because the anxiety phenotype leads to later depression.

## Background

Anxiety and depressive disorders are some of the most common psychiatric diagnoses in children and adolescents respectively. Both are known to be associated with major impairment in childhood and adverse consequences in later life [1-4]. Estimates of the prevalence of any childhood anxiety disorder are in the order of 3 to 12 % [1,4] and rise to as high as 40% or over if impairment is not required for a diagnosis [4]. In general, epidemiological studies show that rates of any anxiety disorder are higher in children than adolescents [5]. In contrast, rates of depressive disorder in young people show higher rates in adolescence (2 to 8 %) than in childhood (1 to 3 %) [6]. Depression and anxiety in childhood and adolescence have long-term deleterious outcomes for a significant proportion of young people. Depression and anxiety, once experienced in childhood are very likely to recur in adulthood [2,7]. Early onset depressive and anxiety disorders are also associated with substantial social impairment [8]. Even sub-clinical levels of depression in children and adolescents are associated with significant morbidity in the form of psychosocial impairment and service utilisation [9,10]. Furthermore, adolescents identified as having high levels of depressive or anxiety symptoms are significantly more likely to experience depressive disorder in adulthood than adolescents with depression levels within the normal range [11,12]. The observations that sub-clinical symptoms of depression are associated with significant morbidity, and that high levels of depression and anxiety symptoms predict depressive and anxiety disorders add to the evidence that depression and anxiety can be regarded as continua [13-15].

Depression and anxiety co-occur more commonly than would be expected by chance in children and adolescents. This co-occurrence has been identified both in clinical studies of children and adolescents and general population samples that have examined sub-clinical levels of depression and anxiety symptoms [16,17]. More specifically, anxiety symptoms or disorders most often precede depressive symptoms or disorders [18-21]. Moreover, although certain sub-types of anxiety, namely social phobia and panic rarely precede depression [22], individuals with these disorders and depression are very likely to have had a different anxiety disorder that predated the onset of depression [20]. Twin studies provide a means of examining the extent to which the genetic and environmental aetiological factors contributing to two disorders or symptom groups overlap and to what extent they are distinct. Longitudinal data, collected at more than one time point provide a further test, namely, that one set of symptoms or disorder is a risk factor for another. This approach is especially useful in the study of anxiety and depression. Several groups have suggested that anxiety may be a developmental precursor of depression, particularly in young people who are at increased risk of depression due to parental depression [23-25]. Indeed Kovacs & Devlin [17] suggested that children may be biologically 'prepared' to experience symptoms of anxiety rather than depression. The results of other studies are consistent with this proposal. For example, in a sample of depressed children, in those children who had a comorbid anxiety disorder, the anxiety disorder was found to have preceded depressive disorder in two thirds of cases [18]. Similar evidence that anxiety disorders tend to precede depression has been reported in longitudinal epidemiological [19,21,26] and clinical studies [20]. Indeed, a recently convened National Institute for Mental Health (NIMH) workgroup recommended research into childhood anxiety as a known precursor of depression as a priority [27]. Despite clear indications that anxiety and depression in childhood and adolescence are associated, it remains unclear as to how the transition from anxiety to depression is mediated over time. Possible factors include aetiological factors in common – these may be 1) genetic or 2) psycho-social risk factors, or 3) a direct risk effect of anxiety leading to later depression.

Cross-sectional twin studies of children [28,29] and adults [30] and one longitudinal twin study of girls [31] have shown that to a large extent, the overlap between anxiety and depression is due to a common set of genes that influence both depression and anxiety. However, shared environmental factors have also been shown to be important sources of covariation between anxiety and depression symptoms for children but not adults [28,31] which suggests the importance of shared psycho-social risk factors for anxiety and depression. Nevertheless, two out of these three twin studies were based on cross-sectional data and were therefore not able to determine the genetic and environmental associations between anxiety and depression over time. Furthermore, despite the importance of understanding why anxiety tends to precede depression, [27] no twin study of children and adolescents has yet specifically tested the hypothesis that anxiety is a phenotypic risk factor for depression. The present study also adds to the existing literature in that data on depression symptoms from different raters (mother and child) are available, thus allowing associations to be examined with data from different informants.

In the present study, we set out to examine two hypotheses that may explain the observed associations between early anxiety and later depression.

1. Early anxiety symptoms and later depression symptoms are associated because of shared risk factors.

2. The association between anxiety symptoms and later depression symptoms is mediated by a risk effect of the phenotype of anxiety.

## Method

### Participants

Families from a systematically ascertained, population-based register of all twin births between 1980 and 1991 in South Wales, U.K. were invited to participate. This register forms a sub-sample of the Cardiff Study of All-Wales and North West of England Twins (CASTANET). Twins who had emigrated were excluded, as were cases in which one of the twins had died or had a serious illness. At the first wave of data collection in 1997, there were a total of 1109 pairs of twins aged 5–17 years although not all of these individuals were eligible to participate at both time points (see below). Data were collected by postal questionnaire. Families received three reminders and telephone reminders when numbers could be traced. The same methods were used three years later to collect longitudinal data except that families received four reminders. To be invited to participate in the follow-up study, we required that the twins were living together in the same home and were under the age of 18 years. Twins were required to live in the same home in order to minimise heterogeneity of environmental risk factors that can impact on genetic and environmental parameter estimates. The focus of the follow-up study was childhood psychopathology and for that reason young people aged 18 and over were not included. At time 1, there were 986 twin pairs who were within the age range of the study at both time points.

In the first wave of the study (1997; time 1), 670 families provided questionnaire responses giving a response rate of 61%. Comparison of responders and non-responders using Townsend Scores [32] which index the level of deprivation of an electoral area revealed no significant socio-demographic differences between the two groups at time 1 (t = .373, p = 0.709). Families with children aged 8–17 were re-contacted in 2000 (time 2). Of the 670 families who replied at time one, 85 had moved away, there were 8 new contraindications and there were 123 children who were out of the age range of the study and did not live in the same home. This left a total of 454 families who were eligible at time 2.

Of these, 338 families replied, giving a total response rate of 75%. There were no significant socio-demographic differences between responders and non-responders at time 2 (t = 1.71, p = 0.09). Zygosity was assigned using a twin similarity questionnaire which has been shown to be over 90% accurate in distinguishing identical (monozygotic; MZ) from fraternal (dizygotic; DZ) twins [33]. There were 198 MZ girls (99 pairs), 134 MZ boys, 128 DZ girls, 116 DZ boys, 270 opposite sex DZ twins.

### Measures

At time 1, parents were asked to complete the Children's Revised Manifest Anxiety Scale [34] which assesses symptoms over the past three months. It has previously been found to be a reliable and valid instrument [35] (Cronbach's α = .8662 twin 1, α = .8708 twin 2). Parents also completed the general functioning scale of the McMasters Family Assessment Device (FAD) [36]. At both time points, parents completed the short version of the Mood and Feelings Questionnaire (MFQ) [37]. At the second wave children aged 11 or above also completed the MFQ. The MFQ is based on DSM-III-R symptoms of depression and has been successfully used as a screening questionnaire for clinical depression in community populations [38] (α = .9231 twin 1, α = 9320 twin 2).

### Analysis

#### Descriptive statistics

For descriptive statistics, (correlations and mean comparisons), the survey commands in the program STATA [39] were used. These commands take into account the clustering of the data from twin pairs (i.e. each twin pair provides two data points) by likening the twin data to a two-stage cluster design with the twin pairs as the primary sampling unit. Since reliability coefficients can not be calculated using these commands these were presented for first and second-born twins separately.

#### Univariate

Analysing data from twins provides a means of estimating the relative contribution of genetic and environmental effects on individual variation in behaviour. In the basic (ACE) model, variation can arise from three sources: 1) additive genetic effects (A); 2) common environmental effects (C); 3) unique environmental effects (E). Common environmental effects are non-genetic factors that serve to make twins more similar to one another while unique environmental effects are non-genetic factors that uniquely influence one individual within a twin pair and tend to make the individuals in a twin pair different from each other. Model fitting was carried out using the programs Mx [41] and LISREL [42] and continuous measures of anxiety and depressive symptoms were analysed. The significance of the A, C and E parameters can be tested by dropping them from the model and comparing the fit of the reduced model to that of the full model using the χ^2 ^critical value for the number of degrees of freedom gained in the reduced model.

#### Bivariate

**Figure 1:**
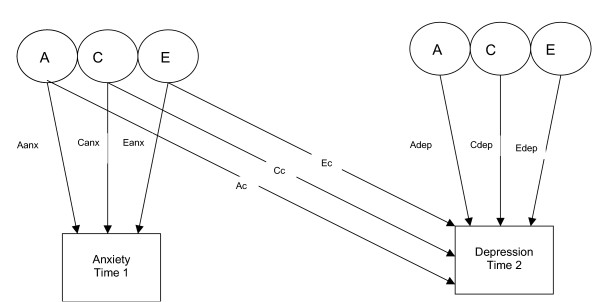
Bivariate Cholesky decomposition of anxiety at time 1 and depression at time 2. Aanx genetic influences on anxiety Canx common environmental influences on anxiety Eanx non shared environmental influences on anxiety Ac genetic influences on anxiety that also influence depression Cc common environmental influences on anxiety that also influence depression Ec non shared environmental influences on anxiety that also influence depression Adep genetic influences specific to depression Cdep common environmental influences specific to depression Edep non shared environmental influences specific to depression

**Figure 2:**
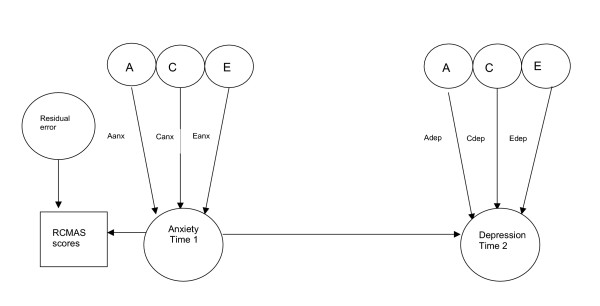
Unidirectional causal model from anxiety at time 1 to depression at time 2 Aanx genetic influences on anxiety Canx common environmental influences on anxiety Eanx non shared environmental influences on anxiety Adep genetic influences specific to depression Cdep common environmental influences specific to depression Edep non shared environmental influences specific to depression

Bivariate analysis allows the covariance of two traits to be partitioned into covariance that is due to additive genetic factors, common environmental factors and unique environmental factors. The covariance parameters for the Cholesky model presented (see figure 1) include those factors in trait 1 (anxiety) that also influence trait 2 (depression). A bivariate model in which anxiety symptoms at time 1 precede depressive symptoms at time 2 was fitted consistent with clinical and epidemiological data showing that anxiety precedes depression more often than vice versa. In addition, a 3 variable model that included anxiety and depressive symptoms at time 1 and depressive symptoms at time 2 was fitted. This model estimated the genetic and environmental associations between anxiety at time 1 and depression at time 2 when the effects of concurrent depression were included. A causal model was then fitted (see figure 2). Comparing the fit of this causal model to that of the general bivariate (Cholesky) model allows two competing explanations of the association between anxiety (time 1) and depression (time 2) to be tested: 1) the association of anxiety and depression is due to genetic and /or environmental risk factors common to both anxiety and depression: 2) the association is due to a risk effect of the phenotype of early anxiety on later depression. A unidirectional causal model from anxiety to depression was fitted given that the data presented are longitudinal. Although the reliabilities of the anxiety and depression scales were good and comparable, a causal model that included residual error was included in line with the suggestion of Neale & Cardon [43]. This was fitted since in direction of causation models it cannot be assumed that measurement error will be confounded with non-shared environmental effects [44]. Fitting this type of model does not constrain measurement error to be transmitted phenotypically and thus is likely to provide more realistic parameter estimates than a casual model without residual error terms.

### Sex effects

Univariate analyses were performed to test for both quantitative and qualitative sex differences. Quantitative sex differences, are tested by estimating the size of parameter estimates for the genders ('the common effects sex limitation model'). Qualitative sex differences test whether the set of genes influencing the phenotype differs by gender i.e. different genes ('the general effects sex limitation model'), this is done by estimating the genetic correlation for opposite sex DZ pairs and comparing the fit of this model to one that constrained the genetic correlation to 0.5. In addition, a bivariate sex limitation model was tested [45]. This model estimates whether the covariation between anxiety and depression is different for boys and girls. All analyses reported were based on the five twin groups (MZ male, MZ female, DZ male, DZ female, DZ opposite sex).

## Results

### Descriptive statistics

There were no significant mean differences on anxiety or depressive symptoms by gender (t = -0.047, p = .962; t = -1.628, p = .104). Age was not associated with anxiety or depressive symptoms (r = .014, p = .707; r = .082, p = .078). The mean age of children at time 1 was 10.58, range 5.58–17.83, and at time 2, was, 12.64, range 8.75–17.25. Symptoms of early anxiety and later depression were strongly correlated (parent-rated symptoms r = .479. The correlation was slightly lower for symptoms across rater (parent rated anxiety and adolescent rated depression), r = .335.

### Sex effects

For anxiety, univariate analysis of parent-rated data showed no significant gender differences in the magnitude of genetic parameter estimates (Δ χ^2 ^= 1.505, Δ df = 3), nor were there qualitative genetic differences (Δ χ^2 ^= 0, Δ df = 1). For depression, univariate models for parent-rated and self-rated scores indicated no significant gender effects for the magnitude of genetic effects (parent rated, Δ χ^2 ^= 2.679, Δ df = 3; self-rated, Δ χ^2 ^= 0.586, Δ df = 3) nor qualitative gender differences (parent rated, Δ χ^2 ^= 0, Δ df = 1; self-rated, Δ χ^2 ^= 0, Δ df = 1). Finally, for parent rated symptoms, results from the bivariate sex limitation Cholesky model showed no significant gender differences in the covariation between anxiety and depression in that the genetic and environmental covariation could be equated across the genders with no significant deterioration in fit (parent-rated, Δ χ^2 ^= 0.445, Δ df = 3). However, for self-rated symptoms, one parameter, i.e., the non-shared environmental covariation parameter, could not be equated across the genders (Δ χ^2 ^= 6.107, Δ df = 1). Estimates from this model for boys were; Aanx = 50, Canx = 17, Eanx = 32, Ac = 10, Cc = 47, Ec = -11, Adep = 9, Cdep = 5, Edep = 40; and for girls were; Aanx = 47, Canx = 22, Eanx = 30, Ac = 12, Cc = 13, Ec = 8, Adep = 30, Cdep = 12, Edep = 24; χ^2 ^= 30.93, df = 32, AIC = -33.07). These results are presented in addition to those for the combined sample (see table 1).

**Table 1:** Bivariate model fitting for time 1 anxiety and time 2 depression

**Rater**	**Aanx**		**Canx**		**Eanx**		**Ac**		**Cc**		**Ec**		**Adep**		**Cdep**		**Edep**		**χ^2^**		**AIC**
Parent rated total sample NMZ = 138 NDZ = 210	46***		24***		30***		13**		36***		2 ns		24*		0 ns		26***		15.36 df = 11		-6.64
Parent rated – adolescents only (8–14 at time 1 and 11–17 at time 2) NMZ = 90 NDZ = 136	46***		23**		31***		11*		34**		4 ns		25*		0 ns		25***		15.02 df = 11		-6.98
Parent rated anxiety time 1 and self rated depression time 2 NMZ = 92 NDZ = 128	53***		15 ns		32***		13*		17 ns		2 ns		36**		0 ns		31***		5.80 df = 11		-16.20

Ns = non significant

* p = .05

** p = .01

*** p = .001

Aanx genetic influences on anxiety

Canx common environmental influences on anxiety

Eanx non shared environmental influences on anxiety

Ac genetic influences on anxiety that also influence depression

Cc common environmental influences on anxiety that also influence depression

Ec non shared environmental influences on anxiety that also influence depression

Adep genetic influences specific to depression

Cdep common environmental influences specific to depression

Edep non shared environmental influences specific to depression

NMZ = number of MZ twin pairs

NDZ = number of DZ twin pairs

Parameter estimates equated to sum 100 in direction of arrows on each trait (anxiety time 1 & depression time 2)

### Bivariate analysis

**Figure 3:**
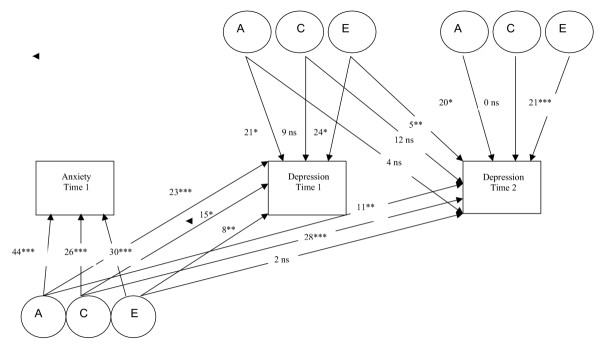
Trivariate Cholesky decomposition of anxiety at time 1, depression at time 1 and depression at time 2 χ^2 ^= 39.74, df = 24, AIC = -8.26 Ns = non significant * p = .05 ** p = .01 *** p = .001 Parameter estimates equated to sum 100 in direction of arrows on each trait (anxiety time 1, depression time 1 & depression time 2)

Table 1 shows results from bivariate analyses of anxiety and depression. Both the genetic covariation (Ac) between anxiety and depression as well as the common environmental covariation (Cc) were significant, while unique environmental covariation (Ec) was negligible. However, despite significant covariation, there were also significant genetic (Adep) and unique environmental effects (Edep) specific to later depression. Thus, although significant genetic covariation between anxiety and depression was observed, the genetic effects on depression were not entirely mediated through genetic effects that were common with anxiety. This illustrates that the genetic covariation between anxiety and depression over time is not complete. Moreover, this observed genetic covariation does not appear to derive from the association of early depression with later depression in that the genetic covariation between anxiety and later depression remained significant when early depressive symptoms were included in the model (see figure 3). Figure 3 shows that there is a significant genetic path linking early anxiety and later depression (Ac _(anx1 dep2) _= 11, p = .001).

It has been shown that the aetiology of depression varies significantly according to age, with greater common environmental effects in children aged 8–10 than in adolescents aged 11–17 [28,46,47]. The three year follow-up period of the study meant that we were unable to analyse children's and adolescent's symptoms separately. However, given previous findings of age effects, we carried out analyses including only those twins who were 'adolescents', that is, aged 11 or over at the second time point with the expectation that the common environmental covariation path (Cc) might decrease. Nonetheless, a significant common environmental component of variance remained.

### Family functioning and rater effects

Following this finding of significant common environmental covariation the nature of this latent factor was further examined. Information on a potential common environmental variable, general family functioning (all twins lived in the parental home) was available. Family functioning was correlated with anxiety symptoms at time 1 (r = .210, p = .001). Bivariate analyses controlling for family functioning are shown in table 2 and it can be seen that this exerted only a slight effect on the estimate of common environmental covariation (drop of Cc from .36 to .31). However, associations between anxiety and depression symptoms could be due to the fact that a single informant (parents) rated their children's anxiety and depression symptoms at both time points. In bivariate analyses, similarities between variables that are due to shared rater effects will be partitioned into the common environmental component of covariance. A distinction should be made here between shared rater effects and rater bias. Rater biases that result in common environmental effects arise when a proxy informant, usually a parent rates a pair of twins as more similar than more objective measures would find them. This sort of rater bias results in deflated MZ phenotypic variance compared to DZ variance [43]. This pattern of variance has not been observed in this sample (anxiety DZ standard error = .035, MZ standard error = .048, t = 1.89, p = .06; depression DZ standard error = .056, MZ standard error = .067, t = .514, p =.608), in fact the MZ variance is greater than the DZ variance. Thus, rater bias can not account for the observed common environmental effects. On the other hand, shared rater effects come about simply as an effect of the same informant rating two sets of symptoms or risk variable and outcome and are therefore not exclusive to parental ratings. In order to test any potential shared rater effects, a bivariate model with data from different informants was tested (parent-rated anxiety symptoms at time 1 and adolescent-rated depression symptoms at time 2). It can be seen from Table 1 that the common environmental covariance influencing anxiety and depression (Cc) is no longer significant when cross-informant information is used. This suggests that at least a proportion of the Cc estimate observed in analyses that used only parent-rated data is likely to be due to shared rater effects, i.e. that part of the shared environmental covariation is due to the fact that the same informant rated both phenotypes. The observation that when family functioning was included as a measured environmental variable in the analyses of parent-rated information, the common environmental covariance estimate was only slightly reduced is consistent with the possibility that these Cc effects may be due to shared rater effects. However, given the small effect sizes of most single measured risk factors (genetic or environmental) [48], one might not expect single environmental risk factors to account for large proportions of variance. Indeed, risk variables for symptoms of depression and anxiety are likely to have multiplicative effects [48,49]. With this in mind, the same family functioning was also included in a cross-informant model. As can be seen from table 2, including family functioning resulted in a small decrease in the Cc estimate (17 to 14).

**Table 2:** Bivariate model fitting for time 1 anxiety and time 2 depression controlling for measured environmental variables

**Rater**	**Aanx**		**Canx**		**Eanx**		**Ac**		**Cc**		**Ec**		**Adep**		**Cdep**		**Edep**		**χ^2^**		**AIC**
Parent rated total sample -**family functioning at time 1 regressed out**	47***		21**		32***		12**		31**		1 ns		24*		0 ns		26***		12.59 df = 11		-9.41
Parent rated anxiety and self rated depression -**family functioning at time 1 regressed out**	56***		11 ns		33***		14*		14 ns		2 ns		38**		0 ns		33***		6.75 df = 11		-15.25

Ns = non significant

* p = .05

** p = .01

*** p = .001

Aanx genetic influences on anxiety

Canx common environmental influences on anxiety

Eanx non shared environmental influences on anxiety

Ac genetic influences on anxiety that also influence depression

Cc common environmental influences on anxiety that also influence depression

Ec non shared environmental influences on anxiety that also influence depression

Adep genetic influences specific to depression

Cdep common environmental influences specific to depression

Edep non shared environmental influences specific to depression

NMZ = number of MZ twin pairs

NDZ = number of DZ twin pairs

Parameter estimates equated to sum 100 in direction of arrows on each trait (anxiety time 1 & depression time 2)

### Phenotypic "causal" model

Finally, a model that included a direct causal path from anxiety to depression was fitted (see figure 2). This model allows for testing whether the phenotype of anxiety (rather than shared genetic and environmental aetiological factors) was responsible for the observed covariance between anxiety and later depression symptoms. That is, testing whether anxiety (independent of shared genetic and environmental risk factors) is itself an early risk factor for depression. The full causal model provided a significantly poorer fit than the general bivariate model (Δ χ^2 ^= 48.82, Δ df = 1, p < .001). Thus, it does not appear that anxiety leads to depression through direct phenotypic effects, but that, anxiety and depression symptoms are associated over time because they share aetiological factors in common.

## Discussion

This investigation has used a longitudinal, epidemiological and genetically sensitive design to examine two possible explanations of the association between early anxiety and later depression symptoms in children and adolescents: 1) a common genetic/environmental aetiology or 2) a phenotypic risk effect of early anxiety. There was significant genetic covariation between anxiety and later depression. (Moreover, the genetic covariation was not explained by the effects of early depression). This result is consistent with the association between early anxiety and later depression being due to a common genetic aetiology. However, the genetic overlap between early anxiety and later depression was far from complete in that there were significant, separate genetic effects on anxiety and genetic influences specific to later depression. Thus, in this sample, symptoms of anxiety and depression in children and adolescents share only a partly common genetic aetiology.

In addition to genetic covariation, significant shared environmental covariation between anxiety and depression was also observed. It appeared that some of the shared environmental covariation between anxiety and depression observed in parent ratings of anxiety and depression was due to shared rater effects as the common environmental covariation (Cc) was no longer significant when analyses were performed with data across different informants. However, including family adversity as a measured environmental risk factor into a model with data from different informants also resulted in a small decrease in the Cc parameter estimate.

The model that included a direct causal path from early anxiety symptoms to later depression symptoms provided a significantly poorer fit than the general bivariate model. These results suggest that anxiety is not an aetiologically distinct phenotype that is in itself a risk factor for future depression symptoms, but rather that the covariation over time arises from the common genetic and environmental architecture. It should be noted though, that some of the common environmental covariation is likely due to shared rater effects, because we found that this path became non significant in cross-informant analyses.

The present findings are consistent with those of several other twin studies that have reported strong genetic correlations between symptoms of anxiety and depression in children and adolescents [28,29,31] and adults [30] and with a study that found significant genetic effects specific to depression [29]. However, only one of these studies was longitudinal [31] and this included girls only, and none of these studies included information from more than one informant.

### Sex effects

The lack of significant univariate and bivariate gender differences in genetic and environmental parameters estimates for parent reports in the present sample is of interest. The results for self reports of depression are less clear, previous analysis of a larger sample, from which the present sample was drawn, did find sex differences for self rated depressive symptoms as measured by the long version of the Mood and Feelings Questionnaire (MFQ) [47], which were not detected in the present sample. However, a previous cross-sectional analysis of the full time 1 self-rated depression data did not find significant gender differences [54]. The only significant gender difference in the present analysis was for the non-shared environmental covariation between anxiety and self-rated depression. The lack of significant effects for univariate analysis for self-rated depression may be due to the smaller sample size and thus lower power to detect effects in the present sample, or it could be due to the fact that different versions of the MFQ that were used in the present (short version) and a previous analysis (long version) [47]. Moreover, the bivariate Cholesky sex limitation analysis for self-reported depression was likely under-powered as few of the parameter estimates reached statistical significance. The sample size is small for those who provided self-reports (NMZ = 92 and NDZ = 128, see table 1) and it is therefore uncertain how reliable these results are. The non-shared environmental covariation estimate for boys also yields a negative parameter estimate (-.11) (albeit non-significant) which indicates the non-shared environment for anxiety is negatively correlated with the non-shared environment for depression. This finding is difficult to interpret, further suggesting caution in conferring too much confidence to the gender-specific findings in this model. Given the sample size for cross informant models in this study, it is not safe to draw firm conclusions about gender differences in the covariance of anxiety and depression when depression is self rated. This needs to be examined in a larger sample. However, although the prevalence of depression shows gender differences in adolescence, this does not necessarily suggest gender differences in aetiology.

How do the present findings fit with results from family studies? Several family studies of the offspring of depressed parents have found increased rates of anxiety rather than depressive disorders [23,25] and Rende and colleagues [24] found that sibling resemblance for anxiety disorders was increased in the offspring of depressed parents. This familial aggregation of anxiety disorders could be due to common environmental or genetic factors. There is now consistent evidence from cross-sectional and longitudinal twin studies of children and adolescents that this observed familial association between anxiety and depression symptoms has a partly common genetic aetiology.

## Limitations

As mentioned previously, several groups have shown that the aetiology of depressive symptoms differs between children (8–10) and adolescents (11–17) [28,46,47]. The majority, though not all, of the present sample were 'children' aged under 10 (range 5–14) at time 1 and 'adolescents' aged 11 and above (range 8–17) at time 2. Thus, as there is age heterogeneity in aetiology, high levels of effects specific to each time point might be expected such as shown in the present study. Nonetheless, we might not expect to find complete genetic overlap between anxiety and depression. For instance, the genetic liability to depression and antisocial behaviour in children and adolescents has also been shown to overlap [50]. Thus, there may be different developmental pathways to depressive symptoms in adolescence. In addition, previous studies have suggested that gene-environment correlation [51,52] and gene-environment interaction [49,53] involving life events also contribute to genetic variance in adolescent depression and such effects would also be subsumed within the estimate of genetic variance.

## Clinical implications

Anxiety and depressive symptoms are strongly associated over time. This association does not appear to be due to a phenotypic risk effect of early anxiety. Rather, early anxiety and later depression are associated due to a common aetiology. This was primarily a common genetic aetiology although family functioning and a single informant rating on both sets of symptoms also contributed to this association. Some of the common genetic aetiology may act as indirect genetic effects via behaviour (gene-environment correlation and gene-environment interaction).

## Competing interests

The author(s) declare that they have no competing interests.

## Author contributions

AT and FR conceived the paper. FR carried out statistical analysis and wrote the paper. AT and MBM wrote and edited the paper. MBM provided statistical support. All authors read and approved the final manuscript.

## Pre-publication history

The pre-publication history for this paper can be accessed here:


